# Effects of *Boswellia Serrata* Roxb. and *Curcuma longa* L. in an In Vitro Intestinal Inflammation Model Using Immune Cells and Caco-2

**DOI:** 10.3390/ph11040126

**Published:** 2018-11-20

**Authors:** Paolo Governa, Maddalena Marchi, Veronica Cocetta, Bianca De Leo, Philippa T. K. Saunders, Daniela Catanzaro, Elisabetta Miraldi, Monica Montopoli, Marco Biagi

**Affiliations:** 1 Department of Physical Sciences, Hearth and Environment, University of Siena, Via Laterina 8, 53100 Siena, Italy; maddimar@hotmail.com (M.M.); miraldi@unisi.it (E.M.); biagi4@unisi.it (M.B.); 2 Department of Biotechnology, Chemistry and Pharmacy–Department of Excellence 2018–2022, University of Siena, Via Aldo Moro 2, 53100 Siena, Italy; 3 Department of Pharmaceutical and Pharmacological Sciences, University of Padua, Largo Egidio Meneghetti 2, 35131 Padua, Italy; veronica.cocetta@gmail.com (V.C.); daniela.catanzaro@unipd.it (D.C.); monica.montopoli@unipd.it (M.M.); 4 MRC Centres for Inflammation Research and Reproductive Health, University of Edinburgh, 47 Little France Crescent, Edinburgh EH16 4TJ, UK; s1263113@ed-alumni.net; 5 MRC Centre for Inflammation Research, University of Edinburgh, 47 Little France Crescent, Edinburgh EH16 4TJ, UK; p.saunders@ed.ac.uk; 6 Venetian Institute of Molecular Medicine, Via Orus 2, 35129 Padua, Italy

**Keywords:** *Boswellia serrata* Roxb., *Curcuma longa* L., intestinal bowel diseases (IBD), Caco-2, PBMC, HMC-1.1, mast cells, cytokines, trans epithelial electrical resistance (TEER), reactive oxygen species (ROS)

## Abstract

Inflammatory bowel diseases, which consist of chronic inflammatory conditions of the colon and the small intestine, are considered a global disease of our modern society. Recently, the interest toward the use of herbal therapies for the management of inflammatory bowel diseases has increased because of their effectiveness and favourable safety profile, compared to conventional drugs. *Boswellia serrata* Roxb. and *Curcuma longa* L. are amongst the most promising herbal drugs, however, their clinical use in inflammatory bowel diseases is limited and little is known on their mechanism of action. The aim of this work was to investigate the effects of two phytochemically characterized extracts of *B. serrata* and *C. longa* in an in vitro model of intestinal inflammation. Their impact on cytokine release and reactive oxygen species production, as well as the maintenance of the intestinal barrier function and on intestinal mucosa immune cells infiltration, has been evaluated. The extracts showed a good protective effect on the intestinal epithelium at 1 µg/mL, with TEER values increasing by approximately 1.5 fold, compared to LPS-stimulated cells. *C. longa* showed an anti-inflammatory mechanism of action, reducing IL-8, TNF-α and IL-6 production by approximately 30%, 25% and 40%, respectively, compared to the inflammatory stimuli. *B. serrata* action was linked to its antioxidant effect, with ROS production being reduced by 25%, compared to H_2_O_2_-stimulated Caco-2 cells. *C. longa* and *B. serrata* resulted to be promising agents for the management of inflammatory bowel diseases by modulating in vitro parameters which have been identified in the clinical conditions.

## 1. Introduction

Inflammatory bowel diseases (IBDs) are a group of diseases very common in modern society, correlated with strong inflammatory conditions of the colon and small intestine [1]. The actual aetiology of IBDs is still unknown [2]. The incidence of IBDs in the 20th century was thought to be limited to Western countries [3], however, in the 21st century the incidence of IBDs has increased throughout the world, making it an important global disease [4].

IBDs are characterized by uncontrolled immune activation against microorganisms which are present in the gut. Mast cells are thought to be critically involved in IBDs pathogenesis, since they are found just beneath the intestinal mucosal barrier, where they can be activated by microbial antigens. These cells can potentially contribute to IBDs through their effects on immune-regulation [5,6]. Indeed, mast cells have been demonstrated to regulate the intestinal epithelium permeability, to initiate and maintain the inflammatory response and are involved in tissue remodelling [7]. Interestingly, some of the conventional drug used for the management of IBD, such as 5-aminosalycilic acid, corticosteroids and even methotrexate, are considered to be effective, at least in part, by acting on mast cells [8,9,10].

The current pharmacological treatments for IBDs focus on the use of drugs, such as 5-aminosalicylates, corticosteroids, immunosuppressive and biological agents, able to reduce inflammation and related symptoms [11]. However, these drugs present some side effects such as rash, nausea and vomiting, which limit their therapeutic application [12].

Herbal therapies have been used since ancient time to treat a wide variety of diseases [13,14] and can also represent a valid alternative to conventional treatments in IBDs due to their validated effectiveness and better profile of safety.

Among the multiple phytotherapic agents available, *Curcuma longa* L. rhizome (turmeric) and *Boswellia serrata* Roxb. gum resin (boswellia) are considered among the most promising herbal drugs for the management of IBDs [15].

According to the European Medicines Agency, *C. longa* root has a therapeutic indication derived from traditional use for the relief of gastrointestinal disorders [16]. Curcuminoids are able to inhibit lipoxygenases, cyclooxygenases and phospholipases and act on AP-1, STAT and NF-κB pathways [17,18]. Furthermore, an important free radical scavenging activity has been associated with the biological activity of turmeric [19]. 

In 2014, McCann and colleagues showed that curcumin-enriched turmeric extracts were able to increase the activity of an IL-10 promoter variant associated with IBD in human embryonic kidney cells [20]. Moreover, curcumin inhibited the proliferation of splenocytes as well as IL-4 and IL-5 secretion by CD4(+) lymphocytes in a mouse model of chemically-induced colitis [21]. The same group, then, demonstrated that curcumin can attenuate the release of MIP-1α, MIP-2 and IL-1β from colonic epithelial cells and macrophages, as well as the release of IL-8 from neutrophils [22].

According to the World Health Organization, boswellia use for the management of IBDs is supported by clinical use [23]. The anti-inflammatory activity of boswellia is related to the inhibition of lipoxygenases and NF-κB [24]. Furthermore, the inhibition of lipid peroxidation and the increase of superoxide dismutase levels, which contribute to the antioxidant activity of boswellia, were correlated to the intestinal anti-inflammatory effect observed in an in vivo colitis model in rats [25,26].

Despite their biological potential, the clinical use of turmeric and boswellia in IBDs is still limited and controversial because of the lack of registered drugs in many countries and the variability of preparations used in clinical trials. 

These reasons led us to investigate the in vitro effectiveness of two dry extracts of *Curcuma longa* L. rhizome (CUR) and *Boswellia serrata* Roxb. gum resin (BOS) as potential drugs for IBDs, by using an innovative multimodal protocol which evaluated the capacity of these herbal drugs in maintaining the intestinal barrier integrity in inflammatory conditions. In the attempt of better investigating the mechanism of action of Cur and BOS in intestinal inflammation, we also considered their involvement in different inflammatory cell responses, evaluating cytokines and ROS release in human epithelial colorectal cells as well as cytokines modulation in immune cells (i.e., peripheral blood mononuclear cells and mast cells).

## 2. Results

### 2.1. Chemical Analyses of Dry Extracts

Table 1 summarizes the chemical details of CUR and BOS.

Curcumin content in CUR, according to *Ph. Eur. 9th* method, resulted 56.85% ± 2.79%. More accurate HPLC-DAD analyses revealed a good reliability for the colorimetric method of *Ph. Eur. 9th*, since total curcuminoids, expressed as curcumin, were found to be 56.06% ± 0.76%. Curcumin represented 87.48% of total curcuminoids, demethoxycurcumin 10.67% and bisdemethoxycurcumin 1.85%. The phytochemical pattern of CUR, thus, was typical of common commercially available C. longa extract enriched in curcuminoids [27] and curcumin content, compared to other curcuminoids, is higher than in native C. longa roots [28]. Moreover, CUR accomplished the content of curcumin declared by the supplier. 

In this study, total triterpenes content in BOS was analysed by means of a rapid, cheap and validated colorimetric method only for quality control purposes of the studied sample. Total triterpenes in BOS resulted 68.41% ± 3.33% of the extract. Indeed, the same sample (same supplier, same batch) was more accurately analysed by Catanzaro and colleagues [29], who reported that boswellic acids in BOS was 39%, being 11-keto-β-boswellic acid (KBA) the main single constituent (5.02%) and acetyl-11-keto-β-boswellic acid (AKBA) being 2.71%.

Differently form CUR, BOS fulfilled the declared chemical composition only in part, since the claimed 65% boswellic acid titration was indeed represented by total triterpenes measured by colorimetric method, whereas boswellic acids were found to be 39%. This results confirmed the concerns claimed by Mannino and co-workers [30] regarding the actual content of boswellic acids in commercially available Boswellia spp. gum-resin extracts. 

### 2.2. Inflammatory Model on Caco-2, PBMC and HMC-1.1: Cytokines Dosages

Firstly, we verified that, as reported in literature, intestinal inflammation is not related to cytokine release by intestinal epithelial cells [31]. We used Caco-2 cells as a model of the intestinal luminal epithelium. 

**Figure 1:**
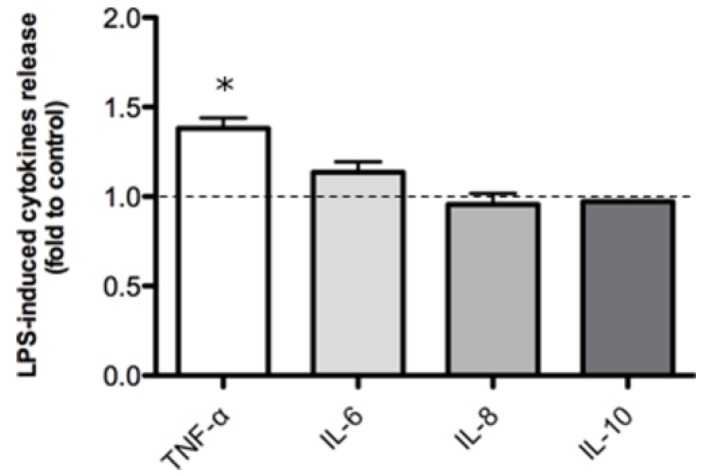
Cytokine release in Caco-2 cells after 24 h of LPS-stimulation (500 ng/mL). The dashed line represent the untreated control. * *p* < 0.05 vs. control.

Measurement of cytokines released by Caco-2, after stimulation with a pro-inflammatory concentration of bacterial lipopolysaccharide (LPS) revealed IL-6, IL-8 and IL-10 levels were not modified by LPS stimulation (500 ng/mL) but TNF-α was upregulated 1.38 fold compared to untreated controls (Figure 1).

Despite the poor release of cytokines by epithelial cells in vitro, cytokine levels are considered pivotal in maintaining and promoting IBDs such as ulcerative colitis and Crohn’s disease. The high levels of cytokine production observed during intestinal inflammation are probably due to infiltrating immune cells [32].

For this reason we focused our attention on a LPS-induced inflammation model using peripheral blood mononuclear cells (PBMC) and we investigated the anti-inflammatory activity of CUR and BOS.

The inflammatory model using PBMC confirmed a significant impact on production of cytokines following LPS stimulation. Notably TNF-α was over than 50 fold up-regulated compared to the control in each experiment we performed. IL-6 also was strongly up-regulated (over 15 folds compared to the control) (Figure S2).

**Figure 2:**
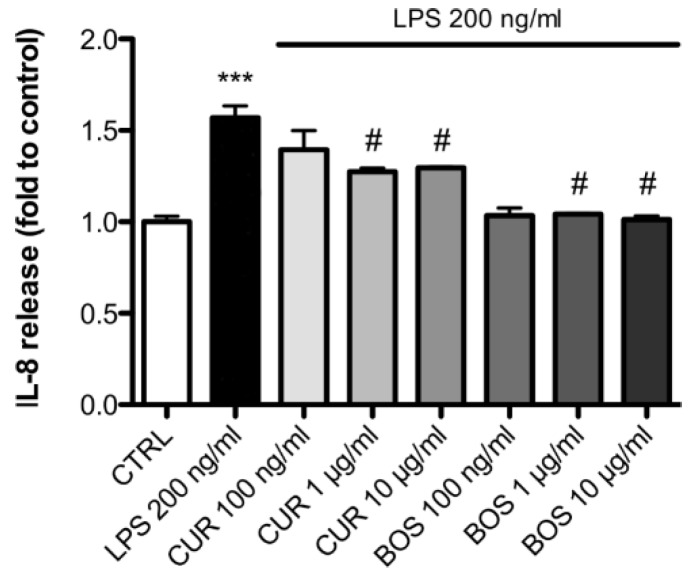
IL-8 release in PBMC after 24 h of LPS stimulation (200 ng/mL); cells were preincubated with CUR and BOS for 24 h. *** *p* < 0.001 vs. control; # *p* < 0.05 vs. stimulus.

IL-8 up-regulation induced by LPS was less striking compared to other cytokines but was statistically significant and confirmed that this chemo-attractant was induced by LPS (Figure 2).

Neither CUR nor BOS at any of the tested concentrations modulated LPS-stimulated TNF-α and IL-6 production by PBMC (Figure S1).

In contrast, IL-8 up-regulation induced by LPS was inhibited by both CUR and BOS (Figure 2); with significant reductions seen with CUR at 1 and 10 µg/mL which inhibited IL-8 release by 14.84% and 13.23%, respectively, compared to LPS-stimulated cells. At the lowest concentration used (100 ng/mL), CUR was not effective in inhibiting IL-8 release in stimulated PBMC.

Likewise, BOS inhibited IL-8 release in stimulated PBMC at each of the tested concentrations, even if statistically significance was obtained only at 1 µg/mL and at 10 µg/mL, with a reduction of at 9.32% and 11.90%, respectively, compared to LPS groups (Figure 2).

**Figure 3:**
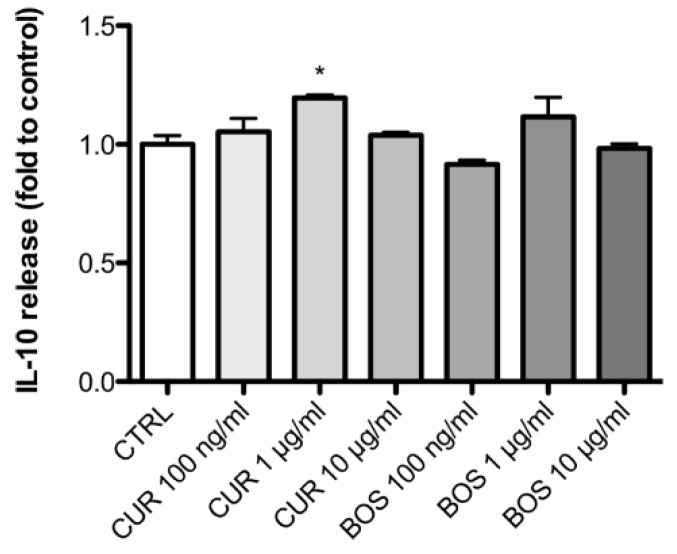
IL-10 release in PBMC after 24 h of incubation with CUR and BOS. * *p* < 0.05 vs. control.

Additionally the release of IL-10, the most representative regulatory cytokine, was monitored in non-inflammatory conditions. IL-10 release by PMBC incubated with CUR was up-regulated by 19.52% compared to the control group. On the contrary, BOS had no effects on IL-10 (Figure 3).

As a result of the preceding experiments with PBMC, the concentration of 1 µg/mL, which was the minimum effective concentration, was chosen for further experiments.

**Figure 4:**
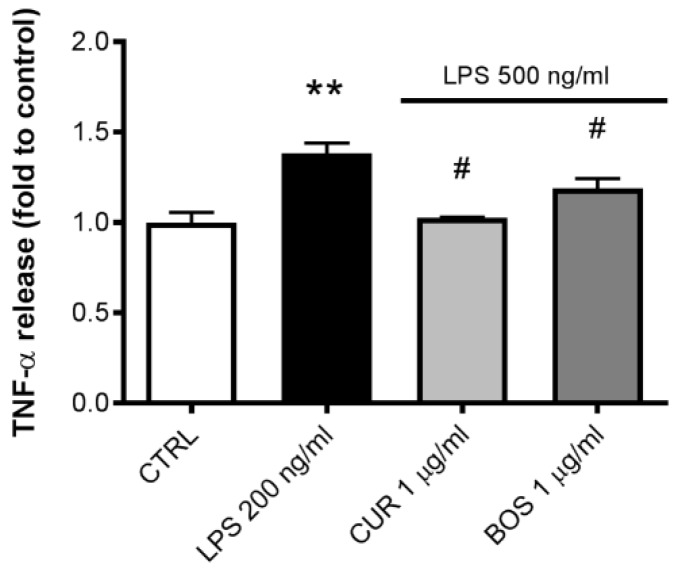
TNF-α release in Caco-2 after 24 h of LPS stimulation (500 ng/mL); cells were preincubated with CUR and BOS for 24 h. ** *p* < 0.01 vs. control; # *p* < 0.05 vs. stimulus.

TNF-α release modulation after LPS stimulation and CUR and BOS treatment on Caco-2 cells was re-evaluated (Figure 4). It was noticeable that incubation with either CUR or BOS was effective in inhibiting LPS-induced TNF-α in Caco-2 by 25.60% and 13.82%, respectively.

Afterwards, in order to better understand the modulation of cytokines observed in PBMC, which are composed of several immune cell subpopulations, we analysed the behaviour of a specific immune cell line involved in IBDs. Hence, we evaluated the release of inflammatory cytokines such as IL-6, IL-8, IL-10 and TNF-α by mast cells (HMC-1.1), as these are believed to play a crucial role in inflammatory conditions.

According to recent literature, TLR4 expression and LPS stimulation in HMC-1.1 cells is controversial [33].Hence, in order to obtain significant and reliable cytokines release, we choose phorbol 12-myristate,13-acetate (PMA) as the inflammatory stimulus [34,35].

Stimulation of HMC-1.1 cells with PMA resulted in increased secretion of TNF-α, IL-6 and IL-8 by 4, 8 and 4,5 folds, respectively, compared to the untreated control.

**Figure 5:**
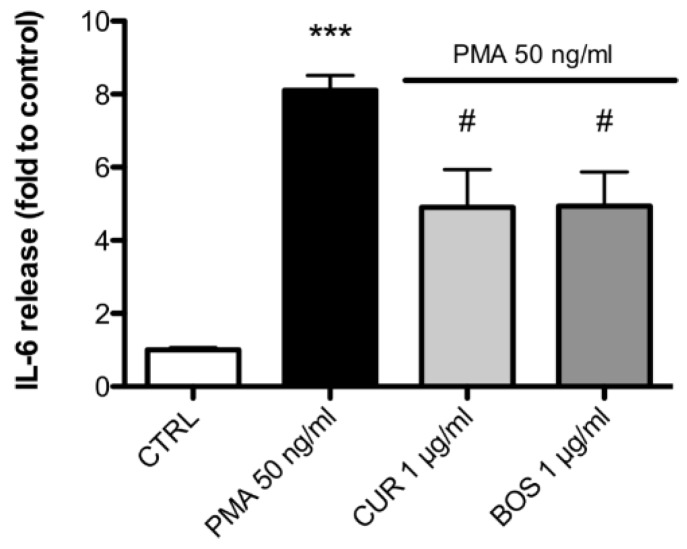
IL-6 release in HMC-1.1 after 24 h of PMA stimulation (50 ng/mL); cells were preincubated with CUR and BOS for 24 h. *** *p* < 0.001 vs. control; # *p* < 0.05 vs. stimulus.

Interestingly, IL-6, the most abundant PMA-induced cytokine produced by HMC 1.1 was significantly inhibited by both CUR and BOS, by 39.44% and 39.06%, respectively (Figure 5). 

Neither CUR nor BOS at each of the tested concentrations modulated the release of TNF-α and IL-8 in HMC-1.1 stimulated by PMA (not shown).

IL-10 release was monitored too but no variation was detected after the treatment of HMC-1.1 cells with CUR and BOS (not shown).

### 2.3. Measurement of ROS Production

Since reactive oxygen species (ROS) production is common in every cell line and it is a typical phenomenon involved in inflammatory conditions [36], we analysed the modulation of ROS by CUR and BOS in Caco-2 cells.

**Figure 6:**
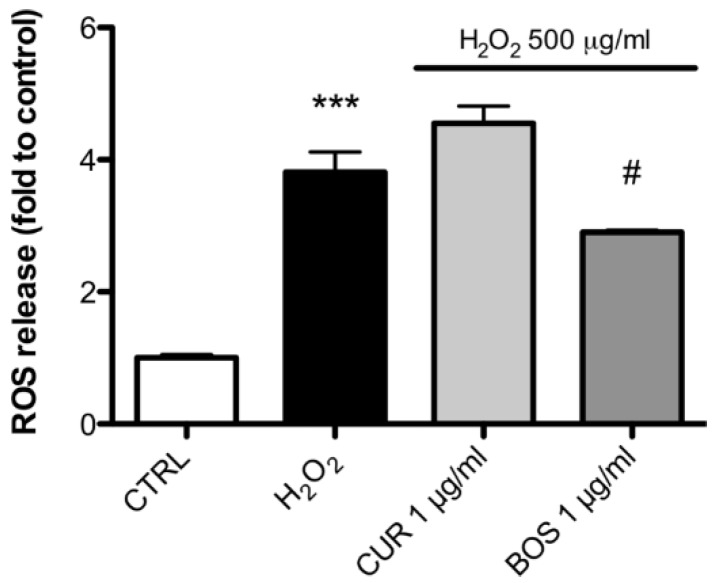
ROS release in Caco-2 cells after H_2_O_2_ stimulation (500 µg/mL); cells were preincubated with CUR and BOS for 24 h. *** *p* < 0.001 vs. control; # *p* < 0.05 vs. stimulus.

Our analysis showed that H_2_O_2_ increased the ROS production in Caco-2 by 3.83 fold compared to the control. BOS 1 µg/mL decreased the ROS production by 24.33% compared to H_2_O_2_, while CUR 1 µg/mL was ineffective in inhibiting ROS production after H_2_O_2_ stimulation (Figure 6).

### 2.4. Intestinal Permeability: TEER Measurements

**Figure 7:**
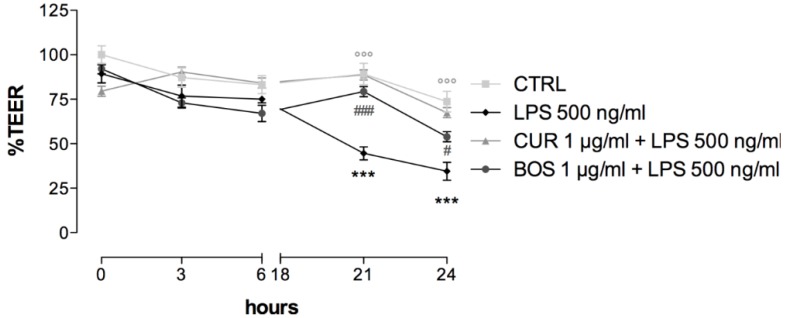
Trans epithelial electrical resistance (TEER) measurement in Caco-2 cells after LPS-stimulation (500 ng/mL). cells were preincubated with CUR and BOS for 24 h. *** *p* < 0.001 stimulus vs. control; °°° *p* < 0.001 CUR vs. stimulus; ### *p* < 0.001 BOS vs. stimulus; # *p* < 0.05 CUR vs. stimulus.

As described before, high levels of cytokines and leukocyte activation could be better framed as a consequence of an alteration in intestinal mucosa integrity and, thus, of increased epithelial permeability. Hence, we evaluated the integrity of the intestinal epithelium barrier by measuring the trans-epithelial electrical resistance (TEER) in LPS-stimulated Caco-2 cells, in order to assess the protective effect of BOS and CUR (Figure 7).

TEER is expressed as the percentage of resistance, normalized to the initial value.

The treatment with LPS decreased the TEER value by 55.04% and 65.51% compared to the not treated control, after 21 and 24 h, respectively.

CUR almost completely protected the intestinal barrier, increasing the TEER value by 43.73% after 21 h and by 32.98% after 24 h, compared to LPS.

The pre-treatment with BOS enhanced the TEER value by 34.33% and 19.41%, after 21 and 24 h, respectively, compared to LPS, thus, significantly reverting the epithelial damage caused by the inflammatory stimulus.

### 2.5. Leukocytes Infiltration: PBMC Adhesion Assay

Immune cell infiltration is a critical event for the establishment of intestinal inflammation. By developing a co-culture between LPS-stimulated Caco-2 and PBMC, we tried to simulate intestinal inflammation, evaluating the effect of CUR and BOS pre-treatment in a qualitative fashion.

**Figure 8:**
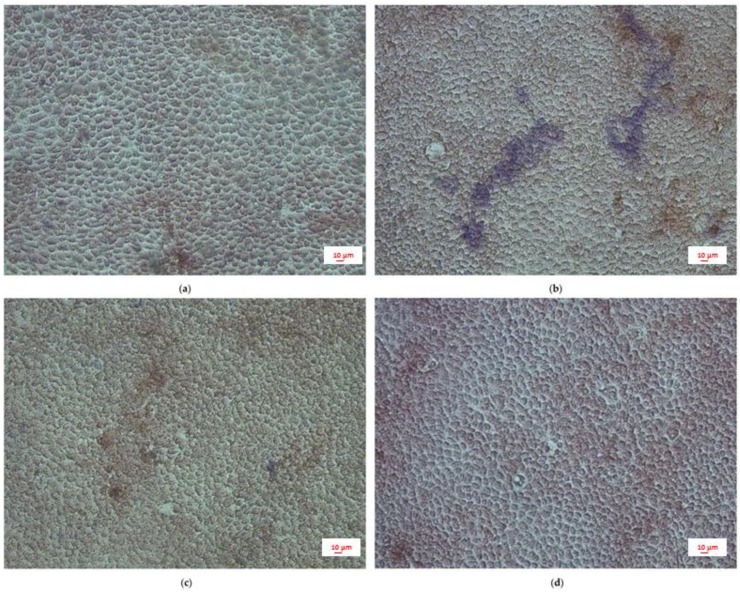
PBMC adhesion assay: (**a**) control, (**b**) LPS 500 ng/mL, (**c**) CUR 1 µg/mL + LPS 500 ng/mL, (**d**) BOS 1 µg/mL + LPS 500 ng/mL. Caco-2 cells were preincubated with CUR and BOS for 24 h and then stimulated with LPS (500 ng/mL) for 24 h. PBMC were co-cultured with Caco-2 for 1 h.

LPS stimulation resulted in a readily detectable leukocyte infiltration, represented by the blue stained spots (Figure 8b), that was inhibited by the treatment with CUR or BOS (Figure 8c,d). 

In agreement with the TEER measurements, the adhesion assay demonstrated a strong protective effect of CUR and BOS on intestinal epithelium.

## 3. Discussion

IBDs are strictly correlated with intestinal barrier dysfunctions, which lead to a strong antigenic response, characterized by oxidative damage and inflammation [37].

In this work, we studied the biological activities of two among the most promising herbal drugs for IBDs management, namely *C. longa* and *B. serrata* [15], by means of an integrated in vitro protocol which considered multiple cell and molecular alterations related to intestinal inflammatory conditions.

CUR and BOS both demonstrated a strong protective effect on the epithelial barrier by increasing TEER values and reducing immune cells infiltration. These data are in agreement with our previous experiments for boswellia [29] but also suggest a novel interesting biological effect of turmeric.

The co-culture of Caco-2 cells and PBMC resulted in a dynamic and more realistic picture of bowel inflammatory conditions, by showing how blood mononuclear cells participate in the infiltration process linked to modified integrity of inflamed intestinal epithelial cells.

In our model, apart from a slight increase in TNF-α levels, we could not detect a change in cytokine levels in LPS-stimulated Caco-2 compared with the non-treated control. These data are consistent with those obtained by Van De Walle and colleagues [38] who observed no effect on cytokines release and other inflammatory marker in LPS-stimulated (10 µg/mL) Caco-2 cells. Nevertheless, Huang and co-workers [39] reported an increase in IL-8 release after 24 h of inflammatory stimulation by using LPS at the concentration of 100 µg/mL. Indeed, intestinal inflammation appears not to be related to cytokine release by epithelial cells. In Caco-2, this is possibly due to the low level of TLR-4 and MD-2 expressed [40]. These receptors, in fact, are mainly expressed at the crypts rather than at the lumen and mediate the response to the LPS stimulus, resulting in NF-κB signal activation [31]. The differences between the experimental results in different studies may be due to the culture conditions and the type of cell clone used [38,41,42]. On the contrary, immune cells are largely responsible for intestinal cytokines accumulation in IBDs [43]. For these reasons, we used immune cells in order to simulate an inflammatory environment, with the aim of better understanding the anti-inflammatory mechanism(s) of action of CUR and BOS in IBDs.

The comprehensive analysis of cytokines modulated by CUR and BOS after inflammatory stimuli on the different cell types revealed that the tested samples had some peculiar anti-inflammatory effectiveness at the concentration of 1 μg/mL. In particular, both CUR and BOS were effective in reducing IL-6 release from mast cells, CUR was more effective in reducing TNF-α release in Caco-2, whereas BOS was more effective in reducing IL-8 release in PBMC. The reduction in IL-8 release from PBMC confirmed the results obtained by Larmonier and colleagues, who observed a similar activity in neutrophils, even if in our model we obtained different results on IL-1β [22].

TNF-α is known to increase intestinal permeability both in vivo and in vitro [44]. The mechanisms by which this occurs are related to the modulation of MLCK expression at the transcriptional level, involving NF-κB pathway [45,46,47]. The effects on MLCK is related to a modulation of claudin-2 expression, which is mediated by the PI3K/Akt pathway and is responsible of the decrease in TEER [48]. Other pathways involved in the TNF-α mediated modulation of intestinal permeability include tyrosine kinases and PKA [49]. Finally, the apoptotic effect of TNF-α has been related to the increase in intestinal permeability [50,51], even if this has been observed only in T84 and HT29/B6 cells but not in Caco-2 cells [52,53,54].

The role of IL-6 in intestinal barrier dysfunctions has been debated [55]. Tazuke and colleagues reported the alteration of intestinal permeability through changes in intracellular phospholipids in IL-6-stimulated Caco-2 cells [56]. Moreover, IL-6 caused a decrease in TEER and tight junctions permeability by stimulating the expression of claudin-2 through the MEK/ERK and PI3K pathways [57]. However, Wang and co-workers observed an increase in keratin-8 and keratin-18 in IL-6-stimulated Caco-2, thus suggesting a protective role of IL-6 in compromised intestinal barrier [58]. More recently, Al-Sadi and colleagues clarified that IL-6 can cause a size-selective (i.e., for molecules having molecular radius < 4 Å) increase in intestinal permeability by stimulating JNK and consequently activating AP-1, which is responsible for claudin-2 increase and TEER decrease [59].

IL-6 production in PMA-stimulated HMC-1 cells has been widely reported [60,61,62]. The intracellular pathways related to cytokines production in PMA-stimulated mast cells include ROS generation and the activation of p38 and NF-κB [62,63].

In HT29 cells, TNF-α stimulated the release of IL-8 through the activation of ERK and p38 [64]. IL-8 is crucial for the recruitment of neutrophils to the lamina propria, even if other chemotactic factors are necessary for the complete transepithelial migration [65]. Moreover, in LPS-stimulated PBMC, the increase in IL-8 levels has been considered a consequence of the increased production of other cytokines, such as TNF-α., through NF-κB and PI3K/Akt activation [66,67].

Regarding IL-10, a protective effect from interferon-γ induced TEER decrease in T84 cells has been reported [68]. In Caco-2, IL-10 protected from TNF-α-induced TEER decrease, when used in combination with glucocorticoids and this effect was related to the activation of the p38 MAPK and the increase in E-cadherin and desmoglein levels [69].The protective effect of IL-10 on intestinal barrier can be due to the induction of heat shock proteins [70] and is associated to the modulation of ZO-1, E-cadherin and occludin in vivo [71]. 

The effects of turmeric and curcumin on cytokines release has been well documented using a plethora of cell and animal models and may be mediated through the modulation of AP-1, MAPK, PKC, MMP and, particularly, NF-κB [72,73,74]. Moreover, curcumin may interact with LPS signalling by downregulating TLR expression, by inhibiting TRL4 dimerization and by binding to the LPS binding site in MD-2 [75]. The involvement of MAPK and NF-κB in inhibiting cytokines release has been also demonstrated in PMA-stimulated mast cells [76].

In this work, we also observed an increase of IL-10 in CUR-treated PBMC, which is consistent with the results obtained by McCann and collaborators [20]. The modulation of IL-10 by turmeric extracts has been recently reviewed [77] and is considered pivotal for the amelioration of IBD symptoms [74,78].

Similarly, boswellia extracts have been demonstrated to reduce cytokines production by inhibiting NF-κB nuclear translocation and this effect seems to be mainly due to boswellic acids [79]. Nevertheless, the reduction of iNOS expression, as well as p38 and JNK (but not ERK) phosphorylation in LPS-stimulated PBMC was attributed to 12-ursene-2-diketone [80].

In the light of these data, we assume that the modulation of the biosynthesis and release of cytokines, although important and significant, cannot actually be considered as the main mechanism of action of these phytotherapics in IBDs.

The antioxidant properties of BOS, rather than its anti-inflammatory effects, may better explain the protective effect of this extract in intestinal inflammation and could be, partly at least, marked as a specific mechanism of action, as previously reported [29]. Indeed, a reduction of lipid peroxidation, together with the increase of superoxide dismutase, catalase, glutathione peroxidase and reduced glutathione were reported in different in vivo models [25,26,80]. In this work, we observed a protective effect of BOS in H_2_O_2_-stimulated Caco-2, which was due to the reduction of ROS production. CUR was not able to protect Caco-2 cells from H_2_O_2_-induced ROS accumulation, even if turmeric is thought to possess an important radical scavenging activity [19].

The inhibition of leukocytes infiltration, along with permeability maintenance and cytokine and ROS level reduction, observed with CUR and BOS treatment, revealed the beneficial effect of these phytotherapics on intestinal inflammation, which occurs through multiple mechanisms of action. Moreover, comparing our findings with previously reported data [29] and preliminary investigations conducted in our laboratories (data not shown), we observed no statistically significant difference between the activity of BOS and CUR compared to of acetyl-11-keto-β-boswellic acid (AKBA) and curcumin (the main constituents of boswellia and turmeric, respectively). This confirms that using the whole herbal phytocomplex, which may have an high content of AKBA and curcumin, can lead to similar therapeutic effects but may that this may also be achieved by administrating a lower concentration of active principles.

## 4. Materials and Methods

### 4.1. Extracts Preparation and Chemical Analysis

A commercial dry extract of *Curcuma longa* L. rhizome, standardized to contain 45% curcumin (Curcuma Phyto Plus, Cento Fiori, Forlì, Italy) was bought in a pharmacy in Siena. Curcumin and curcuminoids content were quantified according to the spectrophotometric method described in the European Pharmacopoeia (*Ph. Eur. 9th*) [7].

The dry extract of *Boswellia serrata* Roxb. gum resin, standardized to contain 65% boswellic acids was gently provided by EOS, Treviso, Italy. Experiments were conducted in triplicate. All the chemicals and solvents were purchased from Sigma-Aldrich, Milan, Italy.

#### 4.1.1. Curcumin Quantification

60 mL of glacial acetic acid were added to 100 mg of CUR and the solution was heated at 90 °C for 60 min under reflux. 2 g of boric acid and 2 g of oxalic acid were added and the solution was heated at 90 °C for 10 min under reflux.

The solution was cooled to room temperature and volume adjusted to 100 mL with glacial acetic acid. 1 mL of the solution was diluted to 50 mL with glacial acetic acid and absorbance recorded at 530 nm using a multiplate spectrophotometer UV-MC2 (Safas, Monaco, Principality of Monaco). Plastic cuvettes (1 cm optical path length) were used.

Curcumin content was calculated according to *Ph. Eur. 9th* as follows:

Curcumin % = (absorbance × 0.426)/weight (g) × dilution factor

#### 4.1.2. Curcumin Quantification (HPLC-DAD Method)

CUR (400 mg) was dissolved in 80 mL of methanol. An aliquot of the solution was diluted 5 folds and filtered through 0.45 μm membrane and used for HPLC analysis.

Analysis of curcuminoids content in CUR were performed using a Shimadzu Prominence LC 2030 3D instrument. A Bondapak^®^ C18 column, 10 µm, 125 Å, 3.9 mm × 300 mm (Waters Corporation, Milford, MA, USA) was used as stationary phase. The mobile phase was composed of water with 0.5% formic acid (A) and acetonitrile with 0.5% formic acid (B) and the gradient phases were: 50% A to 45% A in 8 min, then isocratic phase until 10 min. The flow was 0.9 mL /min and the injected volume was 10 μL.

Absorbance was recorded at 424 nm and quantification of curcumin, demethoxycurcumin and bisdemethoxycurcumin in the extract calculated as curcumin according to the calibration curve obtained using analytical grade curcumin.

#### 4.1.3. Total Triterpenes Quantification

Boswellic acids are correctly quantified by HPLC but in this work, considering the recent analysis performed at University of Padua [29], we confirmed the declared content of boswellic acids using the rapid colorimetric assay published by Fan and He (2006) for total triterpenes [81].

BOS was used as a 10 mg/mL ethanolic solution (96% V/V). 10 µl of the sample solution were added to 190 µl glacial acetic acid. 300 µl of a 5% m/V vanillin in glacial acetic acid solution were added and mixed for 30 s, before adding 1 mL of concentrated perchloric acid.

The mixture was heated at 70 °C for 40 min and, after cooling, the volume was adjusted to 5 mL.

Absorbance was recorded at 548 nm and quantification of total triterpenes in the extract calculated according to the calibration curve obtained using analytical grade ursolic acid.

### 4.2. Cell Cultures

#### 4.2.1. Peripheral Blood Mononuclear Cells

Peripheral blood mononuclear cells (PBMC) were isolated from blood obtained from 3 different healthy volunteers by density gradient centrifugation, using Histopaque^®^-1077 (Sigma-Aldrich, Milan, Italy) [82]. Briefly, Histopaque and freshly collected heparinized blood were added in a 15 mL tube (ratio 3:5) and spun at 325 G for 15 min. Lymphomonocytes ring was recovered in a 15 mL tube and diluted to volume with saline solution. Up to three wash were performed by spinning at 240 G for 10 min, removing supernatant, resuspending cell pellet and diluting to volume with saline solution. PBMC were suspended in a culture medium consisting of RPMI 1460 medium with 1% l-glutamine and 1% penicillin/streptomycin solution. Cell counting was performed using a haemocytometer by Trypan blue staining and the PBMC were used immediately for experiments. Incubation times were conducted at 37 °C, with 5% CO_2_.

#### 4.2.2. Mast Cells

HMC-1.1 were used for experiments on mast cells [83]. An aliquot of HMC-1.1 cells had previously been generously gifted by Dr Butterfield to PTKS allowing us to conduct in vitro mast cell investigations. Cells were cultured in IMDM medium (Gibco, Paisley, UK) supplemented with 10% calf serum (Hyclone, Logan, UT, USA), using 162 cm^3^ cell culture flasks (Corning Incorporated, Costar^®^, Corning, NY, USA) and incubated at 37 °C with 5% of CO_2_. Cells were split and collected in 6 wells culture plates (Corning Incorporated, Costar^®^, Corning, NY, USA) at a density of 1 × 10^6^ cells/mL. Each well contained 3 mL of cell suspension. The HMC-1.1 cells were maintained in IMDM medium, supplemented with 10% calf serum, plus α thioglycerol (Sigma, Gillingham, UK). Cell counting was performed using Countess II Life Technologies Cell Counter (Countess™ Invitrogen, Waltham, MA, USA), by Trypan blue staining.

#### 4.2.3. Intestinal Epithelium Cells

Caco-2 cells were used as a stable in vitro model for the intestinal epithelium [84]. Cells were obtained from the American Type Culture Collection (Manassas, VA, USA) and cultured in DMEM supplemented with 10% foetal bovine serum (FBS), 1% glutamine and 1% penicillin/streptomycin antibiotic (Sigma-Aldrich, Milan, Italy). Cells were maintained under a humidified atmosphere of 5% CO_2_ in air, at 37 °C [85,86].

### 4.3. Evaluation of the Anti-Inflammatory Activity

An in vitro inflammation model was set up for each cell line.

Samples were solubilized in ethanol 85% V/V (Sigma-Aldrich, Milan, Italy) and then diluted in cell culture medium at the concentration of 100 ng/mL, 1 μg/mL and 10 μg/mL, according to preliminary conduced cell viability tests (data not shown).

PBMC (1 × 10^6^ cells/mL) were seeded in 24 well plates and stimulated with LPS from Gram—(from *Salmonella enteridis*, Sigma-Aldrich, Milan, Italy) at a concentration of 200 ng/mL. 

HMC-1.1 (1 × 10^6^ cells/mL) were seeded in 24 well plates and stimulated with PMA (Sigma-Aldrich, Milan, Italy) at a concentration of 50 ng/mL.

Caco-2 (4 × 10^4^) were seeded in 24 well plates, cultured to reach the confluence and stimulated with LPS (500 ng/mL).

A pre-treatment of 24 h with BOS or CUR was administered before the stimulation.

After the incubation time, samples were frozen (at −80 °C) and thawed for 3 times and the supernatants were collected for analysis.

Non-competitive sandwich ELISA kit (Biolegend e-Bioscience DX Diagnostic, Monza, Italy) were used for the dosages of TNF-α, IL-6, IL-8, IL-10, following the procedure reported in the datasheet. Absorbance was recorded at 450 nm using a SAFAS MP96 spectrophotometer. 

### 4.4. Measurement of ROS Production

ROS were quantified using 2′,7′-dichlorofluorescin-diacetate (H_2_-DCF-DA, Sigma-Aldrich, St. Louis, MO, USA), as previously described [17]. Upon cleavage of the acetate groups by intracellular esterase and oxidation, the H_2_−DCF-DA is converted to the fluorescent 2′,7′-dichlorofluorescein (DCF). Briefly, the intestinal cells were seeded into 96-well plates and allowed to adhere overnight. ROS level was measured after the exposure to BOS (1 µg/mL) or to CUR (1 µg/mL) for 24 h. Treatments were removed and H_2_DCF-DA was added to obtain a final concentration of 50 µM in each well. The plate was incubated for 30 min at 37 °C and washed with phosphate-buffered saline (PBS). DCF fluorescence intensity was measured at excitation 485 nm–emission 535 nm, using VICTOR^TM^X3 Multilabel Plate Reader (PerkinElmer, Waltham, MA, USA), before and after the addition of 500 µM H_2_O_2_ on each well.

### 4.5. Measurement of Trans-Epithelial Electric Resistance (TEER)

The efficiency of the barrier functions was evaluated by measuring TEER using a voltmeter [29]. 

Caco-2 cells were placed in Transwell polyester membrane cell culture inserts (transparent PET membrane: 0.4 μm pore size; BD Falcon) as previously described [29]. Culture media was replaced every day.

The integrity of the cell monolayers were monitored by measuring the trans-epithelial electric resistance of the monolayer at confluence from day 14° to day 20° since cells were seeded. When a stable value was reached, a pre-treatment of 24 h was done adding BOS (1 μg/mL) and CUR (1 μg/mL) at the apical chamber in the appropriate wells. 

TEER measurements were performed in HBSS (Hanks’ Balanced Salt solution, Lonza, Milan, Italy) after an equilibration period at room temperature [87,88]. Only cells with TEER value within 360–500 Ω × cm^2^ were used for the experiments [89,90,91].

Treatments were added to the apical chamber and inflammatory stimulus (LPS 500 ng/mL) to the basal chamber. Millicell^®^ ERS meter, Millipore Corporation (Bedford, MA, USA) connected to a pair of chopstick electrodes was inserted in the donor and receiver chambers and the TEER variation at 3, 6, 21 and 24 h after the stimulation was recorded. 

TEER was expressed as percentage of resistance, normalized to initial value.

### 4.6. Adhesion Assay

A simulation of inflammation-induced leukocytes infiltration on intestinal mucosa was performed developing a cell-cell adhesion assay of PBMC to Caco-2 cells, slightly modifying the method published by Seo and colleagues [92]. Haematoxylin-eosin combination was chosen as a simple and efficient staining method [93].

Briefly, glass cover slips (VWR, Italy) were preliminary washed in ethanol (Sigma Aldrich, Milan, Italy), sterilised using a flame, covered with gelatine (Sigma-Aldrich, Milan, Italy) and incubated for 30 min.

Afterwards the gelatine in excess was removed and Caco-2 cells were seeded at the density of 1.5 × 10^5^ cells/well.

Once they reached confluence, cells were pre-treated with BOS (1 μg/mL) and CUR (1 μg/mL) for 24 h and then exposed to LPS 500 ng/mL for 24 h.

PBMC (3 × 10^5^) were co-cultured with Caco-2 for 1 h and then washed with PBS.

Images were obtained using eclipse Ti-s (Nikon, Florence, Italy) microscope.

### 4.7. Statistical Analysis

All experiments were performed in duplicate in three independent repetitions (*n* = 6). 

The statistic differences between groups were determined by the analysis of the variance (one way ANOVA). Values are expressed in the range of +/− standard deviation and *p* < 0.05 was considered statistically significant. Graphs and calculations were performed using GraphPrism^®^ (GraphPad Software, La Jolla, CA, USA).

## 5. Conclusions

Currently, a standardized protocol with universal efficacy for the treatment of chronic intestinal diseases does not exist.

Corticosteroids are commonly prescribed during the acute phases of the disease. 5-aminosalicylates, immunomodulatory therapy or anti-TNF α antibodies can be used in the remission phases, as an alternative or in association with corticosteroids.

All these drugs share considerable side effects and a quite low therapeutic compliance. Therefore, the aim of this study was to evaluate new active principles with the perspective of identifying new and effective treatments for IBDs with a good safety profile.

In vitro studies and animal models largely contributed to the current comprehension of the inflammatory mechanisms at intestinal level. Those mechanisms include a loss of membrane integrity, ROS and cytokines accumulation and the hyper-activation of immune system, particularly referring to mast cells.

In the attempt to evaluate new agents for IBDs treatment, the development of inflammatory models which consider all of these elements is mandatory.

In this work, an original in vitro approach, using various human cell lines and innovative but validated methods, was proposed.

Turmeric and boswellia, which are principally characterized by the anti-inflammatory activity and by a wide and not specific anti-oxidant effect, demonstrated to be promising agents for the management of IBDs, by modulating not only the parameters indicative of dysfunction in the in vitro models used (i.e., cytokines release and ROS production) but also the ones identified in the clinical manifestation of IBDs (i.e., loss of intestinal epithelium integrity and immune cells infiltration), at concentration which can be plausibly reached at intestinal level.

Turmeric extract confirmed to possess anti-inflammatory capacity even at low concentrations and revealed itself as an extraordinary protective agent towards the intestinal epithelium integrity. Boswellia extract showed comparable efficacy in the proposed model, exhibiting a lower anti-inflammatory effect which is counterbalanced by the strong anti-oxidant activity. Moreover, given the peculiar mechanism of action of each extract, it is likely that the association between turmeric and boswellia may act in a synergistic fashion, improving the therapeutic effectiveness. Thus, it is hopefully that future studies will be conducted using a mixture of extracts.

This study must be considered only as a starting point in the developing process of these herbal drugs for IBDs. In that respect, it would be necessary to carry out further molecular and pharmacological investigations and to provide extracts and formulation capable of guaranteeing a good bioavailability, which is one of the main complications of these phytotherapics.

## Figures and Tables

**Table 1:** Chemical composition of *Curcuma longa* L. rhizome (CUR) and *Boswellia serrata* Roxb. gum resin (BOS). *Retrieved from Catanzaro et al., 2015.

Sample	Chemical Marker	Method	%
CUR	total curcuminoids	*Ph. Eur. 9th* method	56.85 ± 2.79
	total curcuminoids	HPLC-DAD	56.06 ± 0.76
	curcumin		49.04 ± 0.40
	demethoxycurcumin		5.98 ± 0.11
	bisdemethoxycurcumin		1.04 ± 0.03
BOS	total triterpenes	Colorimetric method	68.41 ± 3.33
	total boswellic acids*	HPLC-MS HPLC-DAD-ELSD	39
	KBA*		5.02 ± 0.09
	AKBA*		2.71 ± 0.09

## Supplementary Materials

The following are available online at http://www.mdpi.com/1424-8247/11/4/126/s1, Figure S1: TNF-α (**a**), IL-6 (**b**) and IL-8 (**c**) release induced by different concentrations of CUR and BOS in PBMC. No inflammatory effect was observed; Figure S2: TNF-α (**a**) and IL-6 (**b**) release induced by different concentrations of CUR and BOS in LPS-stimulated PBMC. No anti-inflammatory effect was observed. ** *p* < 0.01 vs. control; *** *p* < 0.001 vs. control; Figure S3: IL-6 (**a**) and IL-10 (**b**) release induced by CUR and BOS in HMC-1.1 cells. No effect was observed; Figure S4: TNF-α (**a**) and IL-8 (**b**) release induced by CUR and BOS in PMA-stimulated HMC-1.1 cells. No anti-inflammatory effect was observed. ** *p* < 0.01 vs. control; *** *p* < 0.001 vs. control; Figure S5: Effect of CUR and BOS on ROS production in Caco-2 cells; Figure S6: Effect of CUR and BOS on TEER measurements in Caco-2 cells.

## References

[1] Mudter J. (2011). What’s new about inflammatory bowel diseases in 2011. World J. Gastroenterol..

[2] Burisch J., Munkholm P. (2015). The epidemiology of inflammatory bowel disease. Scand. J. Gastroenterol..

[3] Xia B., Crusius J., Meuwissen S., Pena A. (1998). Inflammatory bowel disease: Definition, epidemiology, etiologic aspects and immunogenetic studies. World J. Gastroenterol..

[4] Ng S.C., Shi H.Y., Hamidi N., Underwood F.E., Tang W., Benchimol E.I., Panaccione R., Ghosh S., Wu J.C.Y., Chan F.K.L. (2017). Worldwide incidence and prevalence of inflammatory bowel disease in the 21st century: A systematic review of population-based studies. Lancet.

[5] Chichlowski M., Westwood G.S., Abraham S.N., Hale L.P. (2010). Role of Mast Cells in Inflammatory Bowel Disease and Inflammation-Associated Colorectal Neoplasia in IL-10-Deficient Mice. PLoS ONE.

[6] He S.-H. (2004). Key role of mast cells and their major secretory products in inflammatory bowel disease. World J. Gastroenterol..

[7] Hamilton M.J., Frei S.M., Stevens R.L. (2014). The Multifaceted Mast Cell in Inflammatory Bowel Disease. Inflamm. Bowel Dis..

[8] Marcondes S., Bau E.C., Antunes E., Dietrich C.P., Nader H.B., De Nucci G. (2002). Inhibition of heparin synthesis by methotrexate in rats in vivo. Biochem. Pharmacol..

[9] Goldsmith P., McGarity B., Walls A.F., Church M.K., Millward-Sadler G.H., Robertson D.A. (1990). Corticosteroid treatment reduces mast cell numbers in inflammatory bowel disease. Dig. Dis. Sci..

[10] Fox C.C., Moore W.C., Lichtenstein L.M. (1991). Modulation of mediator release from human intestinal mast cells by sulfasalazine and 5-aminosalicylic acid. Dig. Dis. Sci..

[11] Neurath M.F. (2017). Current and emerging therapeutic targets for IBD. Nat. Rev. Gastroenterol. Hepatol..

[12] Grevenitis P., Thomas A., Lodhia N. (2015). Medical Therapy for Inflammatory Bowel Disease. Surg. Clin. North. Am..

[13] Tsioutsiou E.E., Giachetti D., Miraldi E., Governa P., Magnano A.R., Biagi M. (2016). Phytotherapy and skin wound healing. Acta. Vulnologica..

[14] Governa P., Baini G., Borgonetti V., Cettolin G., Giachetti D., Magnano A.R., Miraldi E., Biagi M. (2018). Phytotherapy in the Management of Diabetes: A Review. Molecules.

[15] Ng S.C., Lam Y.T., Tsoi K.K.F., Chan F.K.L., Sung J.J.Y., Wu J.C.Y. (2013). Systematic review: The efficacy of herbal therapy in inflammatory bowel disease. Aliment. Pharmacol. Ther..

[16] European Medicines Agency EMA Assessment Report on *Curcuma longa* L. Rhizoma. http://www.ema.europa.eu/docs/en_GB/document_library/Herbal_-_Community_herbal_monograph/2010/02/WC500070703.pdf.

[17] Mazieiro R., Frizon R.R., Barbalho S.M., de Alvares Goulart R. (2018). Is Curcumin a Possibility to Treat Inflammatory Bowel Diseases?. J. Med. Food.

[18] Neto F.C., Marton L.T., de Marqui S.V., Lima T.A., Barbalho S.M. (2018). Curcuminoids From Curcuma Longa: New Adjuvants For The Treatment Of Crohn’S Disease And Ulcerative Colitis?. Crit. Rev. Food Sci. Nutr..

[19] Ghosh S., Banerjee S., Sil P.C. (2015). The beneficial role of curcumin on inflammation, diabetes and neurodegenerative disease: A recent update. Food Chem. Toxicol..

[20] McCann M.J., Johnston S., Reilly K., Men X., Burgess E.J., Perry N.B., Roy N.C. (2014). The effect of turmeric (Curcuma longa) extract on the functionality of the solute carrier protein 22 A4 (SLC22A4) and interleukin-10 (IL-10) variants associated with inflammatory bowel disease. Nutrients.

[21] Billerey-Larmonier C., Uno J.K., Larmonier N., Midura A.J., Timmermann B., Ghishan F.K., Kiela P.R. (2008). Protective effects of dietary curcumin in mouse model of chemically induced colitis are strain dependent. Inflamm. Bowel Dis..

[22] Larmonier C.B., Midura-Kiela M.T., Ramalingam R., Laubitz D., Janikashvili N., Larmonier N., Ghishan F.K., Kiela P.R. (2011). Modulation of neutrophil motility by curcumin: Implications for inflammatory bowel disease. Inflamm. Bowel Dis..

[23] World Health Organization (2009). WHO Monographs on Selected Medicinal Plants Volume 4.

[24] Algieri F., Rodriguez-Nogales A., Rodriguez-Cabezas M.E., Risco S., Ocete M.A., Galvez J. (2015). Botanical Drugs as an Emerging Strategy in Inflammatory Bowel Disease: A Review. Mediators Inflamm..

[25] Hartmann R.M., Fillmann H.S., Martins M.I.M., Meurer L., Marroni N.P. (2014). Boswellia serrata has beneficial anti-inflammatory and antioxidant properties in a model of experimental colitis. Phytother. Res..

[26] Hartmann R.M., Morgan Martins M.I., Tieppo J., Fillmann H.S., Marroni N.P. (2012). Effect of *Boswellia serrata* on antioxidant status in an experimental model of colitis rats induced by acetic acid. Dig. Dis. Sci..

[27] Mudge E., Chan M., Venkataraman S., Brown P.N. (2016). Curcuminoids in Turmeric Roots and Supplements: Method Optimization and Validation. Food Anal. Methods.

[28] Chao I.-C., Wang C.M., Li S.P., Lin L.G., Ye W.C., Zhang Q.W. (2018). Simultaneous Quantification of Three Curcuminoids and Three Volatile Components of Curcuma longa Using Pressurized Liquid Extraction and High-Performance Liquid Chromatography. Molecules.

[29] Catanzaro D., Rancan S., Orso G., Dall’Acqua S., Brun P., Giron M.C., Carrara M., Castagliuolo I., Ragazzi E., Caparrotta L. (2015). Boswellia serrata Preserves Intestinal Epithelial Barrier from Oxidative and Inflammatory Damage. PLoS ONE.

[30] Mannino G., Occhipinti A., Maffei M.E. (2016). Quantitative Determination of 3-O-Acetyl-11-Keto-β-Boswellic Acid (AKBA) and Other Boswellic Acids in *Boswellia sacra* Flueck (syn. *B. carteri* Birdw) and *Boswellia serrata* Roxb. Molecules.

[31] Furrie E., Macfarlane S., Thomson G., Macfarlane G.T. (2005). Toll-like receptors-2, -3 and -4 expression patterns on human colon and their regulation by mucosal-associated bacteria. Immunology.

[32] Hausmann M., Kiessling S., Mestermann S., Webb G., Spottl T., Andus T., Scholmerich J., Herfarth H., Ray K., Falk W. (2002). Toll-like receptors 2 and 4 are up-regulated during intestinal inflammation. Gastroenterology.

[33] Sandig H., Bulfone-Paus S. (2012). TLR signalling in mast cells: Common and unique features. Front. Immunol..

[34] Balletta A., Lorenz D., Rummel A., Gerhard R., Bigalke H., Wegner F. (2013). Human mast cell line-1 (HMC-1) cells exhibit a membrane capacitance increase when dialysed with high free-Ca^2+^ and GTPγS containing intracellular solution. Eur. J. Pharmacol..

[35] Li L., Jin G., Jiang J., Zheng M., Jin Y., Lin Z., Li G., Choi Y., Yan G. (2016). Cornuside inhibits mast cell-mediated allergic response by down-regulating MAPK and NF-κB signalling pathways. Biochem. Biophys. Res. Commun..

[36] Mittal M., Siddiqui M.R., Tran K., Reddy S.P., Malik A.B. (2014). Reactive oxygen species in inflammation and tissue injury. Antioxid. Redox Signal..

[37] Cocetta V., Catanzaro D., Borgonetti V., Ragazzi E., Giron M.C., Governa P., Carnevali I., Monica M., Biagi M. (2019). A Fixed Combination of Probiotics and Herbal Extracts Attenuates Intestinal Barrier Dysfunction from Inflammatory Stress in an In vitro Model Using Caco-2 Cells. Recent Pat. Food. Nutr. Agric..

[38] Van De Walle J., Hendrickx A., Romier B., Larondelle Y., Schneider Y.-J. (2010). Inflammatory parameters in Caco-2 cells: Effect of stimuli nature, concentration, combination and cell differentiation. Toxicol. In Vitro.

[39] Huang Y., Li N., Liboni K., Neu J. (2003). Glutamine decreases lipopolysaccharide-induced IL-8 production in Caco-2 cells through a non-NF-kappaB p50 mechanism. Cytokine.

[40] Abreu M.T., Arnold E.T., Thomas L.S., Gonsky R., Zhou Y., Hu B., Arditi M. (2002). TLR4 and MD-2 expression is regulated by immune-mediated signals in human intestinal epithelial cells. J. Biol. Chem..

[41] Bocker U., Yezerskyy O., Feick P., Manigold T., Panja A., Kalina U., Herweck F., Rossol S., Singer M. (2003). V Responsiveness of intestinal epithelial cell lines to lipopolysaccharide is correlated with Toll-like receptor 4 but not Toll-like receptor 2 or CD14 expression. Int. J. Colorectal Dis..

[42] Cario E., Rosenberg I.M., Brandwein S.L., Beck P.L., Reinecker H.C., Podolsky D.K. (2000). Lipopolysaccharide activates distinct signalling pathways in intestinal epithelial cell lines expressing Toll-like receptors. J. Immunol..

[43] Sanchez-Muñoz F., Dominguez-Lopez A., Yamamoto-Furusho J.K. (2008). Role of cytokines in inflammatory bowel disease. World J. Gastroenterol..

[44] Al-Sadi R., Boivin M., Ma T. (2009). Mechanism of cytokine modulation of epithelial tight junction barrier. Front. Biosci..

[45] Ma T.Y., Boivin M.A., Ye D., Pedram A., Said H.M. (2005). Mechanism of TNF-{α} modulation of Caco-2 intestinal epithelial tight junction barrier: Role of myosin light-chain kinase protein expression. Am. J. Physiol. Gastrointest. Liver Physiol..

[46] Ye D., Ma I., Ma T.Y. (2006). Molecular mechanism of tumor necrosis factor-α modulation of intestinal epithelial tight junction barrier. Am. J. Physiol. Gastrointest. Liver Physiol..

[47] Ye D., Ma T.Y. (2008). Cellular and molecular mechanisms that mediate basal and tumour necrosis factor-α-induced regulation of myosin light chain kinase gene activity. J. Cell. Mol. Med..

[48] Mankertz J., Amasheh M., Krug S.M., Fromm A., Amasheh S., Hillenbrand B., Tavalali S., Fromm M., Schulzke J.D. (2009). TNFα up-regulates claudin-2 expression in epithelial HT-29/B6 cells via phosphatidylinositol-3-kinase signalling. Cell. Tissue Res..

[49] Schmitz H., Fromm M., Bentzel C.J., Scholz P., Detjen K., Mankertz J., Bode H., Epple H.J., Riecken E.O., Schulzke J.D. (1999). Tumor necrosis factor-α (TNFα) regulates the epithelial barrier in the human intestinal cell line HT-29/B6. J. Cell. Sci..

[50] Gitter A.H., Bendfeldt K., Schulzke J.D., Fromm M. (2000). Leaks in the epithelial barrier caused by spontaneous and TNF-α-induced single-cell apoptosis. FASEB J. Off. Publ. Fed. Am. Soc. Exp. Biol..

[51] Florian P., Schoneberg T., Schulzke J.D., Fromm M., Gitter A.H. (2002). Single-cell epithelial defects close rapidly by an actinomyosin purse string mechanism with functional tight junctions. J. Physiol..

[52] Bruewer M., Luegering A., Kucharzik T., Parkos C.A., Madara J.L., Hopkins A.M., Nusrat A. (2003). Proinflammatory cytokines disrupt epithelial barrier function by apoptosis-independent mechanisms. J. Immunol..

[53] Marano C.W., Lewis S.A., Garulacan L.A., Soler A.P., Mullin J.M. (1998). Tumor necrosis factor-α increases sodium and chloride conductance across the tight junction of CACO-2 BBE, a human intestinal epithelial cell line. J. Membr. Biol..

[54] Ma T.Y., Iwamoto G.K., Hoa N.T., Akotia V., Pedram A., Boivin M.A., Said H.M. (2004). TNF-α-induced increase in intestinal epithelial tight junction permeability requires NF-kappa B activation. Am. J. Physiol. Gastrointest. Liver Physiol..

[55] Lee S.H. (2015). Intestinal permeability regulation by tight junction: Implication on inflammatory bowel diseases. Intest. Res..

[56] Tazuke Y., Drongowski R.A., Teitelbaum D.H., Coran A.G. (2003). Interleukin-6 changes tight junction permeability and intracellular phospholipid content in a human enterocyte cell culture model. Pediatr. Surg. Int..

[57] Suzuki T., Yoshinaga N., Tanabe S. (2011). Interleukin-6 (IL-6) regulates claudin-2 expression and tight junction permeability in intestinal epithelium. J. Biol. Chem..

[58] Wang L., Srinivasan S., Theiss A.L., Merlin D., Sitaraman S. (2007). V Interleukin-6 induces keratin expression in intestinal epithelial cells: Potential role of keratin-8 in interleukin-6-induced barrier function alterations. J. Biol. Chem..

[59] Al-Sadi R., Ye D., Boivin M., Guo S., Hashimi M., Ereifej L., Ma T.Y. (2014). Interleukin-6 Modulation of Intestinal Epithelial Tight Junction Permeability Is Mediated by JNK Pathway Activation of Claudin-2 Gene. PLoS ONE.

[60] Hsieh C.-J., Hall K., Ha T., Li C., Krishnaswamy G., Chi D.S. (2007). Baicalein inhibits IL-1β- and TNF-α-induced inflammatory cytokine production from human mast cells via regulation of the NF-κB pathway. Clin. Mol. Allergy.

[61] Yano K., Nakao K., Sayama K., Hamasaki K., Kato Y., Nakata K., Ishii N., Butterfield J.H., Galli S.J. (1997). The HMC-1 human mast cell line expresses the hepatocyte growth factor receptor c-met. Biochem. Commun..

[62] Kruger-Krasagakes S., Moller A., Kolde G., Lippert U., Weber M., Henz B.M. (1996). Production of interleukin-6 by human mast cells and basophilic cells. J. Invest. Dermatol..

[63] Kim J.Y., Ro J.Y. (2005). Signal pathway of cytokines produced by reactive oxygen species generated from phorbol myristate acetate-stimulated HMC-1 cells. Scand. J. Immunol..

[64] Jijon H.B., Panenka W.J., Madsen K.L., Parsons H.G. (2002). MAP kinases contribute to IL-8 secretion by intestinal epithelial cells via a posttranscriptional mechanism. Am. J. Physiol. Cell. Physiol..

[65] Kucharzik T., Hudson J.T., Lügering A., Abbas J.A., Bettini M., Lake J.G., Evans M.E., Ziegler T.R., Merlin D., Madara J.L., Williams I.R. (2005). Acute induction of human IL-8 production by intestinal epithelium triggers neutrophil infiltration without mucosal injury. Gut.

[66] DeForge L.E., Kenney J.S., Jones M.L., Warren J.S., Remick D.G. (1992). Biphasic production of IL-8 in lipopolysaccharide (LPS)-stimulated human whole blood. Separation of LPS- and cytokine-stimulated components using anti-tumor necrosis factor and anti-IL-1 antibodies. J. Immunol..

[67] Osawa Y., Nagaki M., Banno Y., Brenner D.A., Asano T., Nozawa Y., Moriwaki H., Nakashima S. (2002). Tumor Necrosis Factor A-Induced Interleukin-8 Production via NF-κB and Phosphatidylinositol 3-Kinase/Akt Pathways Inhibits Cell Apoptosis in Human Hepatocytes. Infect. Immun..

[68] Madsen K.L., Lewis S.A., Tavernini M.M., Hibbard J., Fedorak R.N. (1997). Interleukin 10 prevents cytokine-induced disruption of T84 monolayer barrier integrity and limits chloride secretion. Gastroenterology.

[69] Loren V., Cabre E., Ojanguren I., Domenech E., Pedrosa E., Garcia-Jaraquemada A., Manosa M., Manye J. (2015). Interleukin-10 Enhances the Intestinal Epithelial Barrier in the Presence of Corticosteroids through p38 MAPK Activity in Caco-2 Monolayers: A Possible Mechanism for Steroid Responsiveness in Ulcerative Colitis. PLoS ONE.

[70] Wang Q., Hasselgren P.-O. (2002). Heat shock response reduces intestinal permeability in septic mice: Potential role of interleukin-10. Am. J. Physiol. Regul. Integr. Comp. Physiol..

[71] Sun X., Yang H., Nose K., Nose S., Haxhija E.Q., Koga H., Feng Y., Teitelbaum D.H. (2008). Decline in intestinal mucosal IL-10 expression and decreased intestinal barrier function in a mouse model of total parenteral nutrition. Am. J. Physiol. Gastrointest. Liver Physiol..

[72] Hewlings S.J., Kalman D.S. (2017). Curcumin: A Review of Its’ Effects on Human Health. Foods.

[73] Jurenka J.S. (2009). Anti-inflammatory properties of curcumin, a major constituent of Curcuma longa: A review of preclinical and clinical research. Altern. Med. Rev..

[74] Epstein J., Docena G., MacDonald T.T., Sanderson I.R. (2010). Curcumin suppresses p38 mitogen-activated protein kinase activation, reduces IL-1beta and matrix metalloproteinase-3 and enhances IL-10 in the mucosa of children and adults with inflammatory bowel disease. Br. J. Nutr..

[75] Aggarwal B.B., Gupta S.C., Sung B. (2013). Curcumin: An orally bioavailable blocker of TNF and other pro-inflammatory biomarkers. Br. J. Pharmacol..

[76] Zhang N., Li H., Jia J., He M. (2015). Anti-inflammatory effect of curcumin on mast cell-mediated allergic responses in ovalbumin-induced allergic rhinitis mouse. Cell. Immunol..

[77] Mollazadeh H., Cicero A.F.G., Blesso C.N., Pirro M., Majeed M., Sahebkar A. (2017). Immune modulation by curcumin: The role of interleukin-10. Crit. Rev. Food Sci. Nutr..

[78] Larmonier C.B., Uno J.K., Lee K.-M., Karrasch T., Laubitz D., Thurston R., Midura-Kiela M.T., Ghishan F.K., Sartor R.B., Jobin C. (2008). Limited effects of dietary curcumin on Th-1 driven colitis in IL-10 deficient mice suggest an IL-10-dependent mechanism of protection. Am. J. Physiol. Gastrointest. Liver Physiol..

[79] Ammon H.P.T. (2010). Modulation of the immune system by Boswellia serrata extracts and boswellic acids. Phytomedicine.

[80] Gayathri B., Manjula N., Vinaykumar K.S., Lakshmi B.S., Balakrishnan A. (2007). Pure compound from Boswellia serrata extract exhibits anti-inflammatory property in human PBMCs and mouse macrophages through inhibition of TNFα, IL-1beta, NO and MAP kinases. Int. Immunopharmacol..

[81] Fan J.-P., He C.-H. (2006). Simultaneous quantification of three major bioactive triterpene acids in the leaves of Diospyros kaki by high-performance liquid chromatography method. J. Pharm. Biomed. Anal..

[82] Panda S.K., Ravindran B. (2013). Isolation of Human PBMCs. Bio-Protocol.

[83] Sundström M., Vliagoftis H., Karlberg P., Butterfield J.H., Nilsson K., Metcalfe D.D., Nilsson G. (2003). Functional and phenotypic studies of two variants of a human mast cell line with a distinct set of mutations in the c-kit proto-oncogene. Immunology.

[84] Sambuy Y., De Angelis I., Ranaldi G., Scarino M.L., Stammati A., Zucco F. (2005). The Caco-2 cell line as a model of the intestinal barrier: Influence of cell and culture-related factors on Caco-2 cell functional characteristics. Cell. Biol. Toxicol..

[85] Dall’Acqua S., Catanzaro D., Cocetta V., Igl N., Ragazzi E., Giron M.C., Cecconello L., Montopoli M. (2016). Protective effects of psi taraxasterol 3-O-myristate and arnidiol 3-O-myristate isolated from Calendula officinalis on epithelial intestinal barrier. Fitoterapia.

[86] Cocetta V., Borgonetti V., Ragazzi E., Cecilia Giron M., Catanzaro D., Cecilia M., Governa P., Carnevali I., Biagi M., Montopoli M. (2017). A Fixed Combination of Probiotics and Herbal Extracts Attenuates Intestinal Barrier Dysfunction from Inflammatory Stress. Preprints.

[87] Liu T., Chang L.-J., Uss A., Chu I., Morrison R.A., Wang L., Prelusky D., Cheng K.-C., Li C. (2010). The impact of protein on Caco-2 permeability of low mass balance compounds for absorption projection and efflux substrate identification. J. Pharm. Biomed. Anal..

[88] Vidau C., Brunet J.-L., Badiou A., Belzunces L.P. (2009). Phenylpyrazole insecticides induce cytotoxicity by altering mechanisms involved in cellular energy supply in the human epithelial cell model Caco-2. Toxicol. In Vitro.

[89] Hubatsch I., Ragnarsson E.G.E., Artursson P. (2007). Determination of drug permeability and prediction of drug absorption in Caco-2 monolayers. Nat. Protoc..

[90] Natoli M., Leoni B.D., D’Agnano I., Zucco F., Felsani A. (2012). Good Caco-2 cell culture practices. Toxicol. In Vitro.

[91] Srinivasan B., Kolli A.R., Esch M.B., Abaci H.E., Shuler M.L., Hickman J.J. (2015). TEER measurement techniques for in vitro barrier model systems. J. Lab. Autom..

[92] Seo W.Y., Youn G.S., Choi S.Y., Park J. (2015). Butein, a tetrahydroxychalcone, suppresses pro-inflammatory responses in HaCaT keratinocytes. BMB Rep..

[93] Chan J.K.C. (2014). The Wonderful Colors of the Hematoxylin–Eosin Stain in Diagnostic Surgical Pathology. Int. J. Surg. Pathol..

