# Supplementary figures and images for: Effects of Boswellia Serrata Roxb. and Curcuma longa L. in an In Vitro Intestinal Inflammation Model Using Immune Cells and Caco-2
Source: Pharmaceuticals (Basel). 2018 Nov 20;11(4):126. doi: 10.3390/ph11040126 (PMC6316569; doi:10.3390/ph11040126)

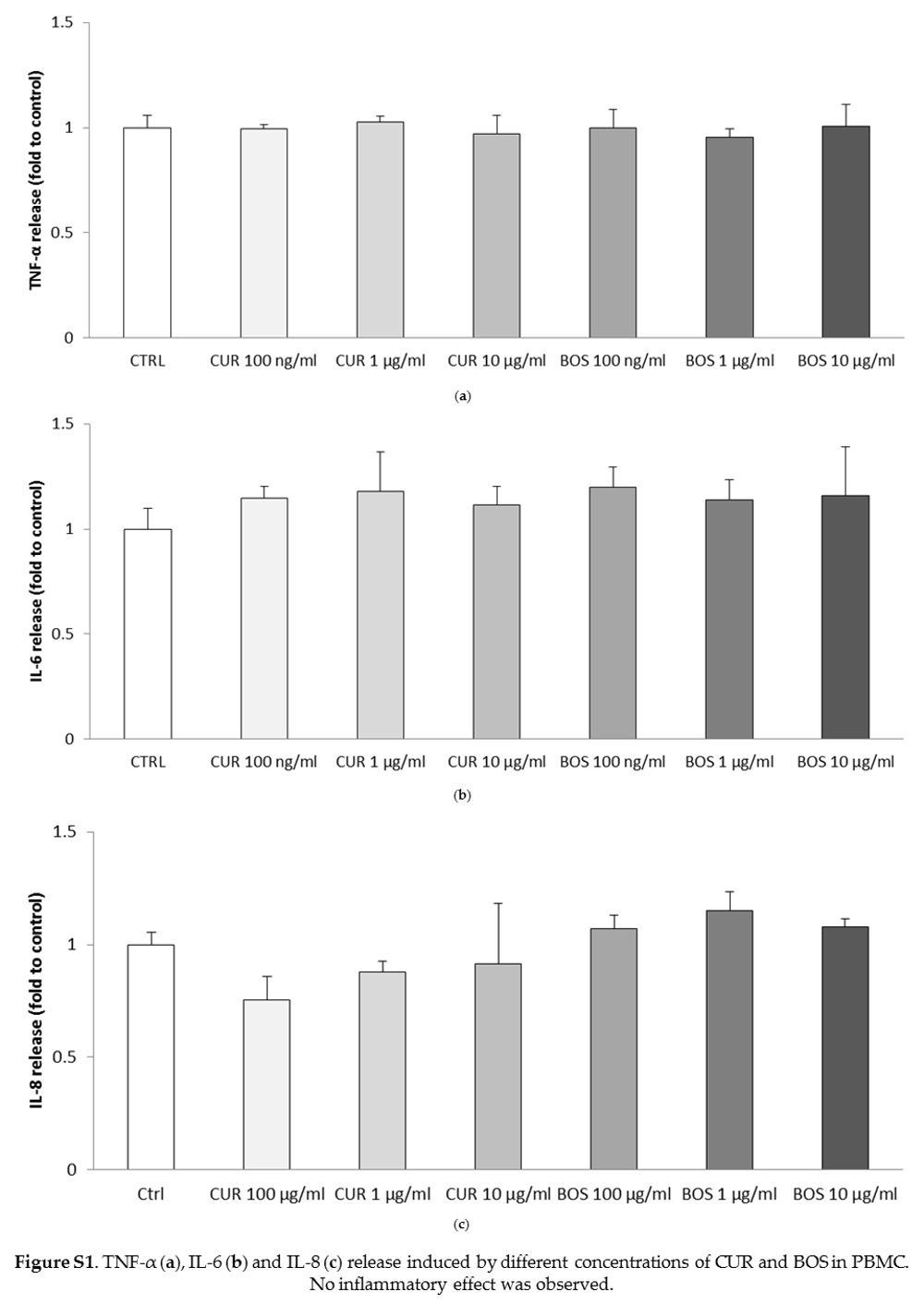

Supplement: Supplementary file 1 [file pharmaceuticals-11-00126-s001.zip › Figure S1.TIF]

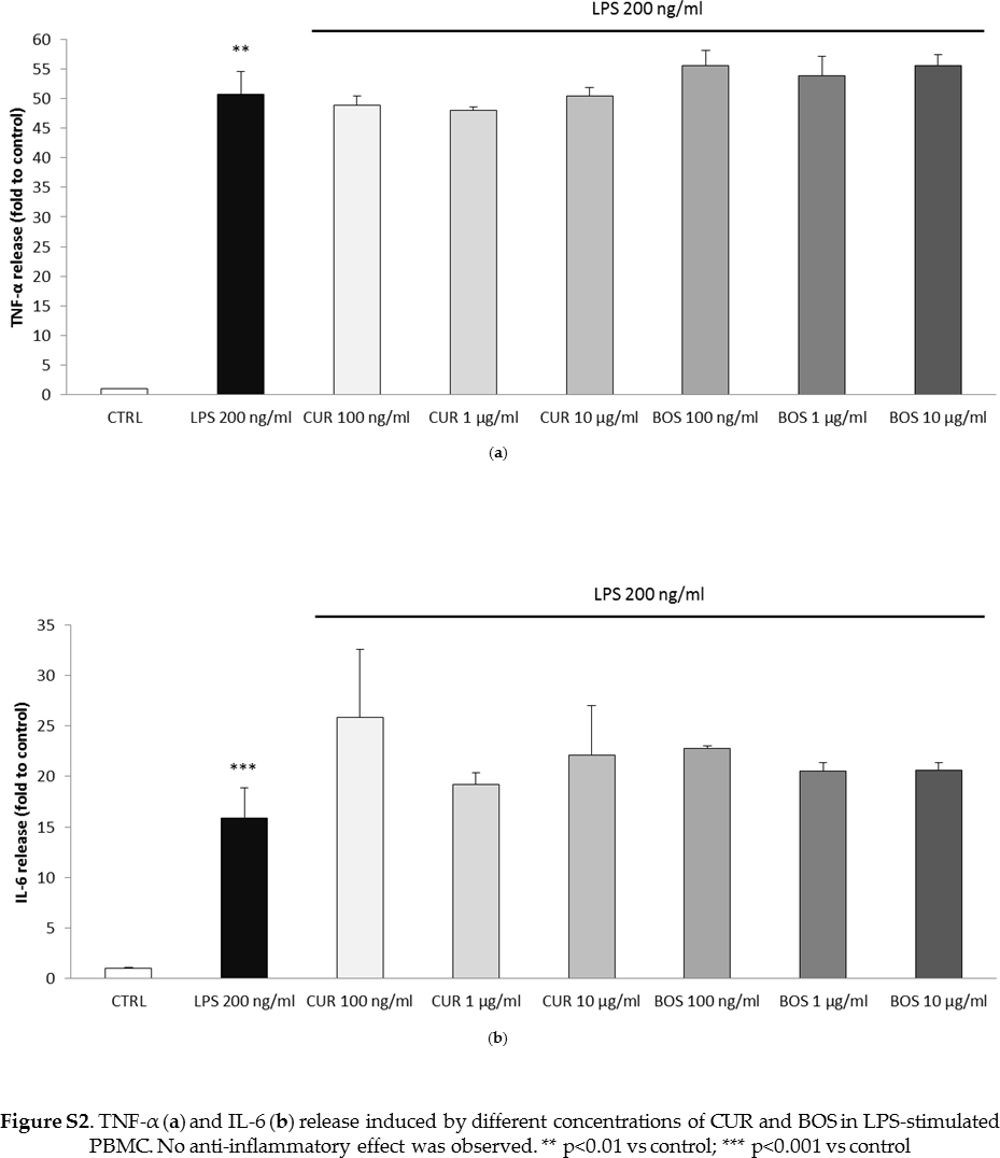

Supplement: Supplementary file 1 [file pharmaceuticals-11-00126-s001.zip › Figure_S2.TIF]

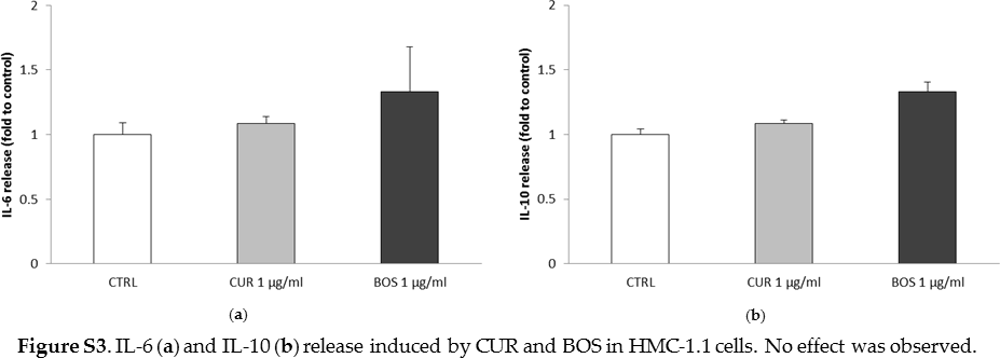

Supplement: Supplementary file 1 [file pharmaceuticals-11-00126-s001.zip › Figure_S3.TIF]

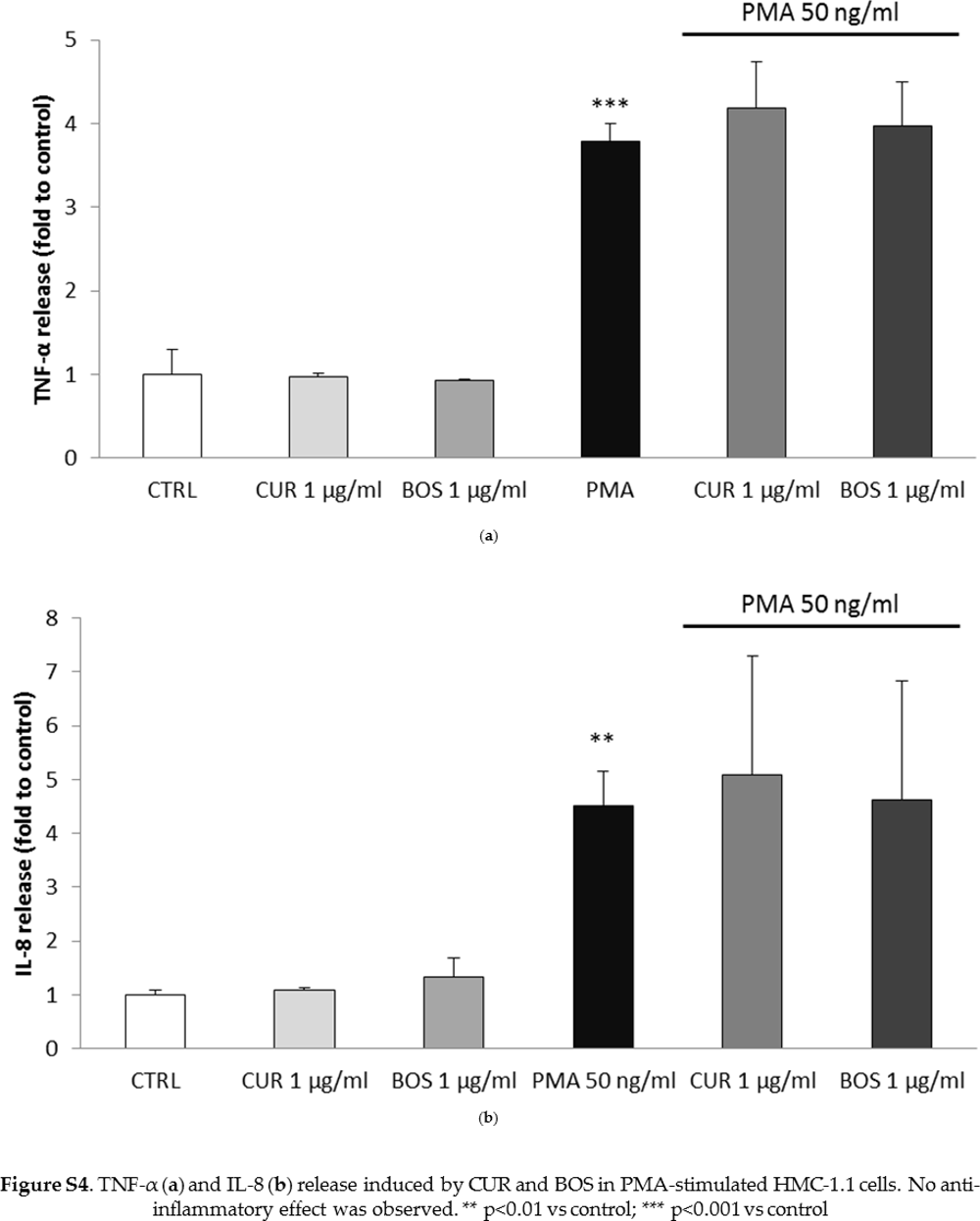

Supplement: Supplementary file 1 [file pharmaceuticals-11-00126-s001.zip › Figure_S4.TIF]

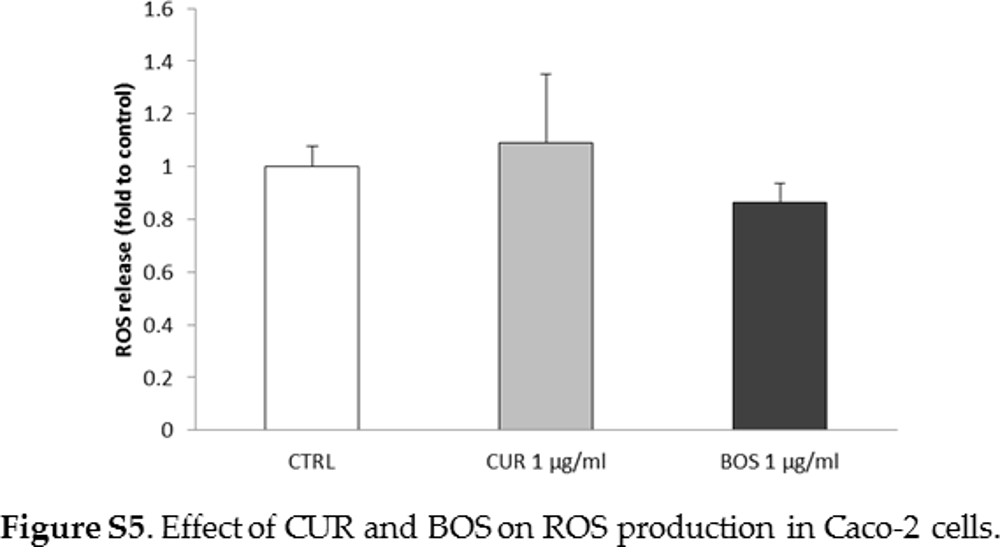

Supplement: Supplementary file 1 [file pharmaceuticals-11-00126-s001.zip › Figure_S5.TIF]

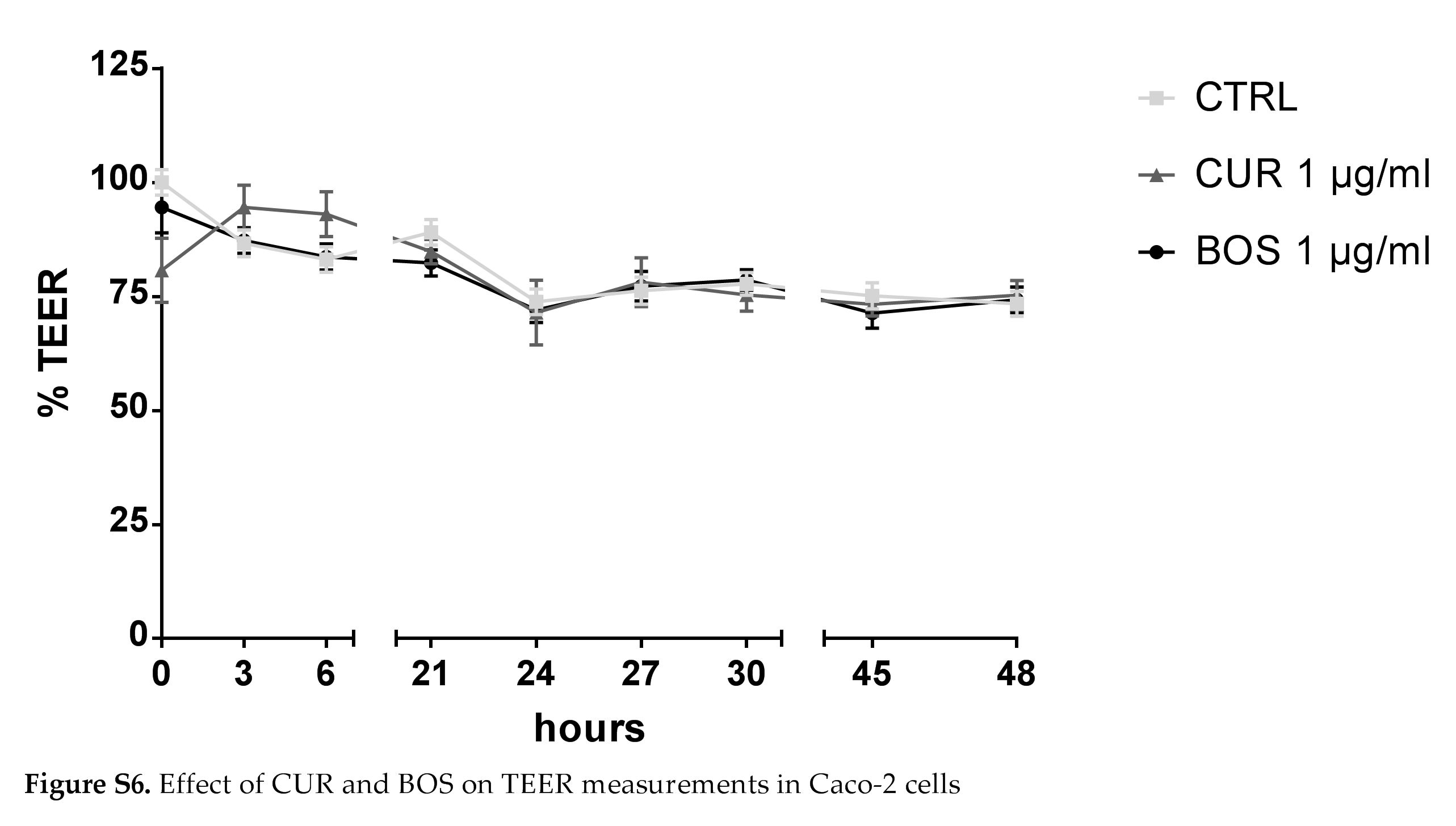

Supplement: Supplementary file 1 [file pharmaceuticals-11-00126-s001.zip › Figure_S6.TIF]
